# Sequential Combination of Microwave- and Ultrasound-Assisted Extraction of Total Flavonoids from *Osmanthus fragrans* Lour. Flowers

**DOI:** 10.3390/molecules22122216

**Published:** 2017-12-13

**Authors:** Jianfeng Yu, Qi Lou, Xiangyang Zheng, Zhengwei Cui, Jian Fu

**Affiliations:** 1 Food Engineering & Machinery Group, School of Mechanical Engineering, Jiangnan University, 1800 Lihu Avenue, Wuxi 214122, China; 18861822453@163.com (Q.L.); 17625010805@163.com (X.Z.); Cuizhengwei@jiangnan.edu.cn (Z.C.); 18352537966@163.com (J.F.); 2 Jiangsu Key Laboratory of Advanced Food Manufacturing Equipment &Technology, 1800 Lihu Avenue, Wuxi 214122, China

**Keywords:** *Osmanthus fragrans* Lour. flower, total flavonoids, microwave-assisted extraction, ultrasound-assisted extraction, response surface methodology

## Abstract

Microwave-assisted and ultrasound-assisted extraction assays were used to isolate total flavonoids (TF) from *Osmanthus fragrans* flowers. The effects of the solid-liquid ratio, ethanol concentration, microwave power, microwave extraction time, ultrasonic power and ultrasonic extraction time on the yield of TF were studied. A sequential combination of microwave- and ultrasound-assisted extraction (SC-MUAE) methods was developed, which was subsequently optimized by Box-Behnken design-response surface methodology (BBD-RSM). The interaction effects of the ethanol concentration (40–60%), microwave extraction time (5–7 min), ultrasonic extraction time (8–12 min) and ultrasonic power (210–430 W) on the yield of TF were investigated. The optimum operating parameters for the extraction of TF were determined to be as follows: ethanol concentration (48.15%), microwave extraction time (6.43 min), ultrasonic extraction time (10.09 min) and ultrasonic power (370.9 W). Under these conditions, the extraction yield of TF was 7.86 mg/g.

## 1. Introduction

*Osmanthus fragrans* Lour., belonging to the Oleaceae family, is cultivated as both an ornamental plant and used as a traditional folk medicine in southern and central China for the treatment of a wide range of diseases [1]. This plant’s flowers are especially valued in China as a common tea beverage and flavour additive for foods, and the essential oil of the flowers is high value added and used to produce various kinds of perfumes.

Flavonoids are naturally synthesized polyphenols that are important constituents of some plants and berries. They have been used as antioxidants and free radical scavengers, enhancing the antioxidant capacity in the human body. The mean daily intake of these secondary metabolites from natural sources for humans is up to 250–500 mg. Total flavonoids (TF) are one bioactive compound known to be important in reported pharmacological effects. TF are exploited various disease-curing usage in anticancer, anti-inflammatory, antitussive, cardioprotective and antitumour effects depending on their antioxidant functions [2]. Since the human body cannot synthesize TF itself, methods for extracting natural TF from plant materials have been analysed in recent years. Microwave-assisted extraction and ultrasound-assisted extraction are the two most prominently used methods in plant compound extraction [3,4,5,6,7].

Microwave-assisted extraction (MAE) is a process that takes advantage of the effects of microwaves to extract biological materials. Localized heating of solid materials causes moisture to rapidly evaporate, which produces tremendous pressure and causes the expansion and rupture of cells, facilitating the release of the desired intracellular contents [8,9,10]. Therefore, this method liberates active compounds into the solvent [11]. MAE has the benefits of faster heating, lower thermal gradients, smaller equipment size and increased extraction yield. MAE is more widely applied for natural compound extraction in contrast to traditional methods. Ultrasound-assisted extraction (UAE) also has been widely applied in solid-liquid extraction as a novel cell disruption method based on the high shear force created by high frequency ultrasound [12,13,14]. This technique is a fast and effective method for the extraction of isoflavones from plants [15]. Ultrasound waves are able to go through a liquid medium or a liquid containing solid materials by causing compression and expansion effects on the plant cells, producing cavitation. Cavitation involves the production of air bubbles, their formation and their collapse. The cavitation phenomenon enhances biomass diffusion, cell disruption and finally solvent penetration. Ultrasound extraction generally provides a better lipid extraction rate than microwave extraction [16]. These techniques have certain insufficiencies, such as the attenuation effect in the case of ultrasound and low penetration depth and uncontrolled heating with the microwave assay [17]. The abovementioned methods are also subject to remarkable shortcomings and limitations such as long extraction times, more solvent consumption and low recovery of purified product solvent extraction [18]. 

Simultaneous irradiation with ultrasound and microwave energy can be conducted to accelerate the extraction process and expedite the release of the targets from the matrix in a short time [19]. Cheng et al. [20] studied a simultaneous ultrasonic and microwave extraction approach to extract the flavonoids in *S. suberectus*. Wu et al. [21] investigated a simultaneous microwave/ultrasonic-assisted enzymatic extraction method to perform antioxidant ingredient extraction from *Nitraria tangutorum* juice by-products.

Qin et al. [22] hypothesized that employing a sequential ultrasound extraction and microwave extraction could potentially minimize or prevent the degradation of the extract. Total flavonoids are phenolic compounds and contain glycosidic bonds that give them relatively poor stability [23]. It is necessary to investigate the effect of a sequential combination of MAE/UAE on extraction yield of bioactive compounds from plant materials but, to date, little research on the effects of the sequential combination of MAE and UAE (SC-MUAE), and the sequential combination of UAE and MAE (SC-UMAE) on the extraction of total flavonoids from *O. fragrans* has been reported in the scientific literature. Furthermore, the optimized conditions of SC-MUAE for the extraction of total flavonoids from *O. fragrans* Lour. flower have not been investigated until now.

In this study, we aimed to systematically investigate the effects of processing parameters, including solid-liquid ratio, ethanol concentration, microwave power, microwave extraction time, ultrasonic power and ultrasonic extraction time, in the process of SC-MUAE for the extraction of total flavonoids from the *O. fragrans* flower. Subsequently, optimization experiments were conducted to obtain the optimized processing conditions of SC-MUAE by Box-Behnken design (BBD) and response surface methodology (RSM).

## 2. Results and Discussion

### 2.1. Effect of Different Extraction Methods on the Physical Structure

**Figure 1:**
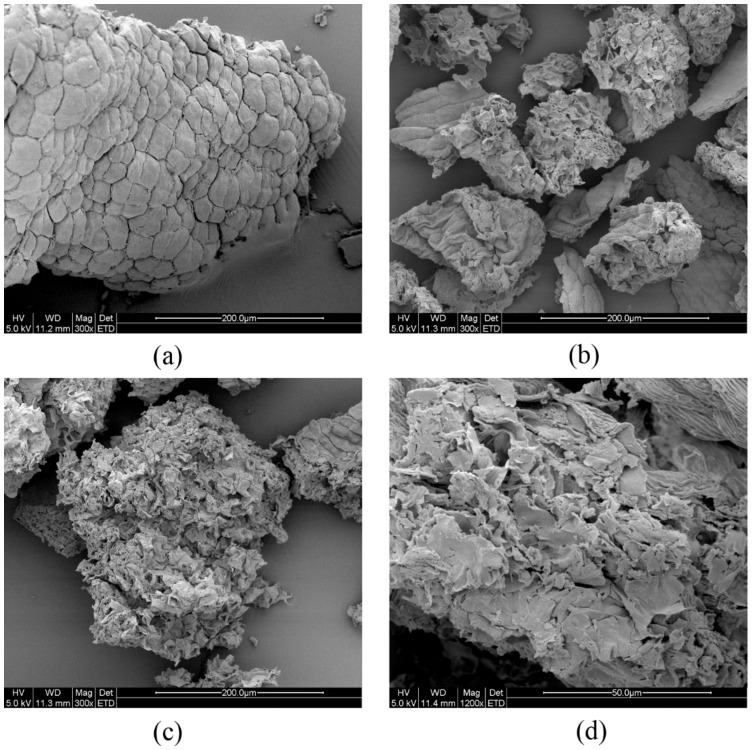
Scanning electron micrographs of cell surfaces: (**a**) Cells before treatment; (**b**) cells with MAE; (**c**) cells with UAE; (**d**) cells with SC-MUAE treatment.

Scanning electron micrographs of four samples are shown in Figure 1. Figure 1a shows the intact cellular wall before treatment. In this case, the original cell structure of *O. fragrans* is smooth with intact cell walls. Figure 1b shows that the cell wall of *O. fragrans* was partially ruptured after MAE treatment. The sample treated by the MAE method exhibits a highly wrinkled surface and perforations of the visible membrane can be seen. The ruptured condition of the cellular wall and membranes can be observed in Figure 1c, where we can observe that UAE has a better effect than MAE on the rupture of cellular tissue. Figure 1d shows the microstructure of ruptured tissue after treatment by SC-MUAE. From Figure 1d, it is shown that the cell structures were disrupted thoroughly and the compounds of the flower can be thoroughly extracted using the SC-MUAE method.

### 2.2. Effect of Solid-Liquid Ratio on Extraction Yield of Total Flavonoids

Preliminary studies were performed in order to investigate the influence of the optimal solid-liquid ratio on extraction yield of total flavonoids while the all other extraction parameters were fixed. The experimental conditions with different solid-liquid ratios are described below:

(1) In the first case, accurately weighed samples (3, 2, 1.5, 1.2, 1, 0.857, 0.75, 0.67, 0.6 and 0.5 g) of flower powder were put into separate conical flasks. Then, 30 mL of 50% (*v*/*v*) aqueous-ethanol solution was added to each flask. The mixture solution was treated in the microwave extractor at a power level of 465 W for 3 min. This extraction process was repeated for three cycles.
(2) In the second case, accurately weighed samples (3, 2, 1.5, 1.2, 1, 0.857, 0.75, 0.67, 0.6 and 0.5 g) of flower powder were put into separate conical flasks. Then, 30 mL of 50% (*v*/*v*) aqueous-ethanol solution was added to each flask. The mixture solution was treated in the ultrasound extractor at a power level of 288 W for 20 min.
(3) In the third case, accurately weighed samples (3, 2, 1.5, 1.2, 1, 0.857, 0.75, 0.67, 0.6 and 0.5 g) of flower powder were put into separate conical flasks. Then, 30 mL of 50% (*v*/*v*) aqueous-ethanol solution was added to each flask. The ten mixture solutions were placed in the ultrasound extractor at a power level of 288 W for 20 min. The ultrasound-treated solutions were then transferred to the microwave extractor and heated at a power level of 465 W for 3 min. The extractions were performed in triplicate.
(4) In the fourth case, accurately weighted samples (3, 2, 1.5, 1.2, 1, 0.857, 0.75, 0.67, 0.6 and 0.5 g) of flower powder were put into separate conical flasks. Then, 30 mL of 50% (*v*/*v*) aqueous-ethanol solution was added to each flask. The ten mixture solutions were placed in the microwave extractor at a power level of 465 W for 3 min. The microwave-treated mixture solutions were then transferred to the ultrasound extractor and treated at a power level of 288 W for 20 min. The extractions were performed in triplicate.

**Figure 2:**
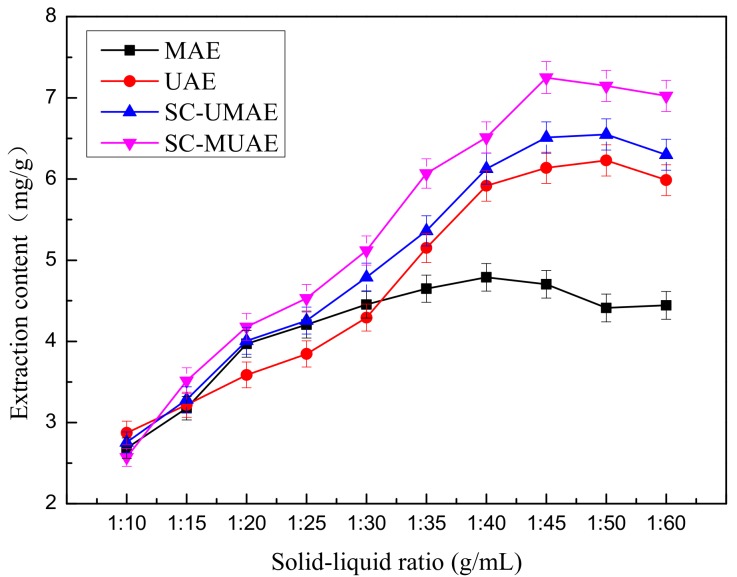
Effect of solid-liquid ratio on extraction content.

The TFC affected by different solid-liquid ratios is shown in Figure 2. It should be noted that the TFC initially increases with the increase of the solid-liquid ratio, reaches its maximum at 1:45, and then begins to decrease. Water and low concentrations of ethanol can easily gain access to cells but high concentrations of ethanol can cause protein denaturation, preventing the dissolution of TF and therefore influencing the extraction rate. Dahmoune et al. [3] found the same phenomenon when polyphenols were extracted from *Myrtus communis* L. leaves by a microwave-assisted method.

Meanwhile, the different effects of the four kinds of extraction methods can be observed in Figure 2. When the solid-liquid ratio ranged from 1:35 to 1:60, the SC-MUAE extraction method has the highest extraction efficiency among SC-UMAE, MAE and UAE. For the SC-MUAE extraction method, when the solid-liquid ratio was 1:45, the maximum extraction content (7.25 mg/g) was obtained. Thus, 1:45 was considered to be the optimal ratio for further experiments.

### 2.3. Effect of Ethanol Concentration on Extraction Yield of Total Flavonoids

**Figure 3:**
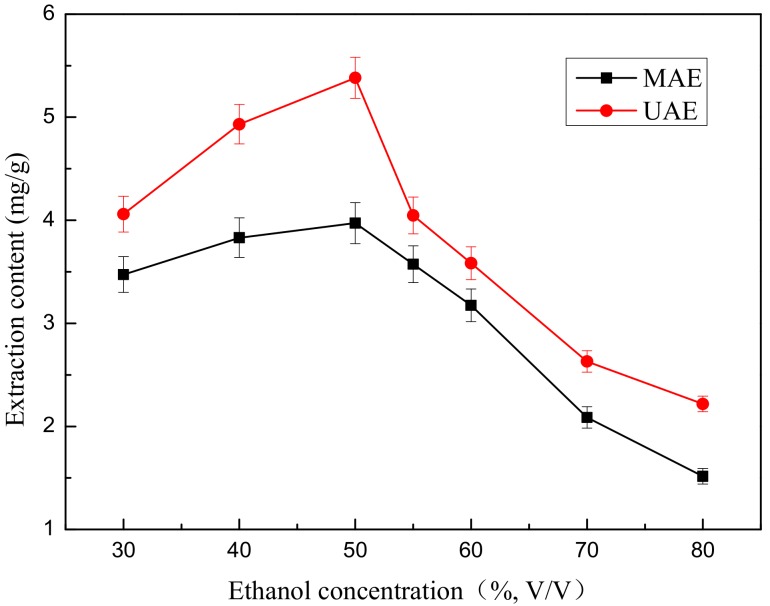
Effect of ethanol concentration on extraction content.

Ethanol concentration is one of the influencing factors for extraction and is an important index for evaluation of extraction efficiency. The effect of ethanol concentration on the extraction content of total flavonoids was studied using the MAE and UAE assays. 0.667 g of ground flower powder was mixed with 30 mL of aqueous-ethanol solutions in different concentrations (*v*/*v*) of 30, 40, 50, 60, 70 and 80%. In the first case, the mixed solutions were treated by microwave with a power level of 465 W for 3 min. In the second case, the mixed solutions were treated by ultrasound with a power level of 324 W for 20 min. The experimental results under different ethanol concentrations are depicted in Figure 3.

In Figure 3, the ethanol concentration has a favourable effect on the extraction content of total flavonoids when the ethanol concentration ranged from 30% to 50% (*v*/*v*), after which the yield of flavonoids decreased with increasing ethanol concentration. When the ethanol concentration was exceeded 70% (*v*/*v*), the extraction yield decreased dramatically. This might be because a higher ethanol concentration may change the solvent polarity and affect the solubility of total flavonoids [24].

The extraction content gradually increases with increasing ethanol concentration and reached a maximum value (5.382 mg/g) when the ethanol concentration ranged from 30% to 50% (*v*/*v*) and then remarkably decreased from 50% to 80% (*v*/*v*). We found that 50% (*v*/*v*) was the optimal ethanol concentration for the extraction process.

### 2.4. Effect of Microwave Power on Extraction Yield of Total Flavonoids

The influence of microwave power on the extraction efficiency was examined by varying magnetron power, while other experimental parameters were held constant for all of the reactions in this set of experiments. 

First, 0.667 g of ground flower powder was weighed and added to 30 mL of 50% (*v*/*v*) ethanol to set the liquid-solid ratio at 1:45. The mixed solution was immersed for 10 min, and the mixture was subsequently treated by the microwave irradiation at various consumption power levels (38, 387, 465, 630, 775, 917 and 1065 W) for 2.5 min. After extraction, the mixture was cooled in a 20 °C water bath for 3 min. The extracted sample was then centrifuged to obtain the supernatant as above. Each test was also repeated in triplicate.

**Figure 4:**
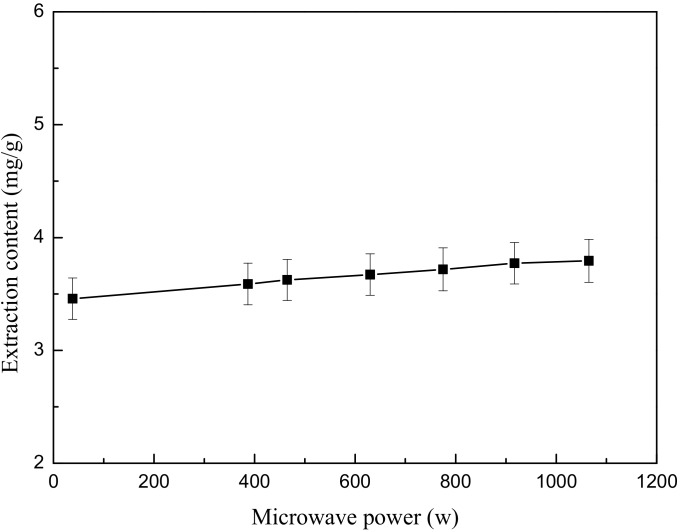
Effect of microwave power on extraction content.

As shown in Figure 4, the content of flavonoids varied from 3.458 mg/g to 3.794 mg/g when the magnetron power ranged from 38 W to 1065 W. It could be observed that increasing microwave power would only slightly promote the dissolution of flavonoid components of the *O. fragrans* flower when the extraction time was kept constant. Most papers report that the increase in microwave power is insignificant [25].

### 2.5. Effect of Microwave Extraction Time on Extraction Yield of Total Flavonoids

Microwave extraction time is another significant factor that could influence the extraction efficiency for total flavonoids. In this study, the effects of different microwave extraction times on the yield of total flavonoids were evaluated while other extraction parameters were fixed. 

The first case: 0.667 g weight of ground flower powder was added to 30 mL of 50% (*v*/*v*) ethanol. The second case: 0.667 g weight of ground flower powder was added to 30 mL of deionized water. After the powder soaked in the liquid for 10 min, the mixed solution was treated by microwave extractor at a power level of 465 W for different treatment times (3, 4, 5, 6, 7 and 8 min). After extraction, the mixture was cooled in a 20 °C water bath for 3 min. Then, centrifugation was carried out to obtain the supernatant as above. Each test was also repeated in triplicate.

**Figure 5:**
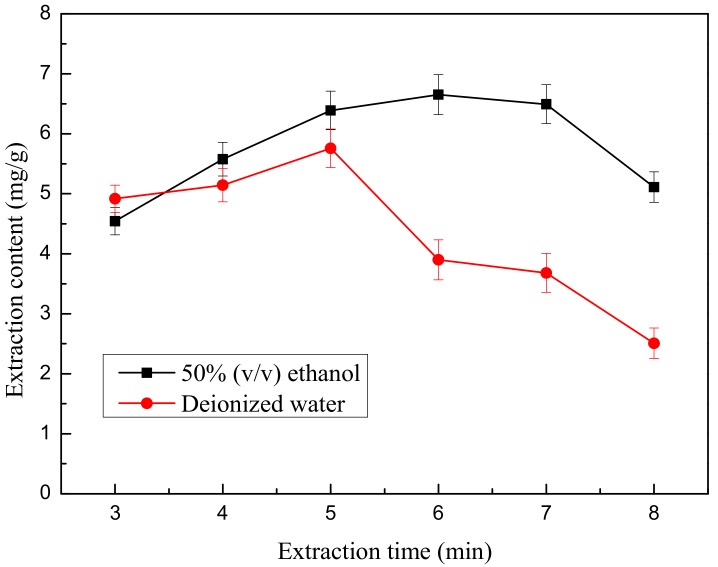
Effect of different microwave treatment times by different solvents on microwave extraction.

Figure 5 reveals the effect of differing microwave extraction times (3–8 min) on the yield of flavonoids. The variance was relatively rapid when extraction time varied from 3 min to 7 min and reached a maximum value of 6.793 mg/g at 7 min. When microwave treatment time ranged from 3 min to 7 min with 50% (*v*/*v*) aqueous-ethanol as the solvent, the elevation of TF increased comparatively. After the 6 min mark, TF yield no longer markedly increased with a further extension of microwave extraction time. Eventually, the flavonoid yield decreased slowly with increasing time, indicating that flavonoids might be more rapidly dissolved or the flavonoids might decompose with the further increasing extraction time. Therefore, a microwave extraction time of 6 min was selected as the central point for RSM.

It has been found that higher absorbance of microwave energy by polar molecules such as TF can result in higher solution temperatures, which leads to the decomposition of the extracted components and may even cook the sample [26]. This may be a reason for the decrease in TFC of the extracts from this study at longer microwave extraction times. In the experiment, when the microwave extraction time reached 7 min, the mixture in the beaker was boiling, which indicates that the flavonoids are due to degradation.

### 2.6. Effect of Ultrasonic Power on Extraction Yield of Total Flavonoids

First, 4.444 g weight of ground flower powder was added to 200 mL of 50% (*v*/*v*) ethanol. After the powder soaked in the liquid for 10 min, the mixture was treated by ultrasound extractor at six different ultrasonic power levels (108, 216, 324, 432, 540 and 648 W) for 5 min. After extraction, the mixture was cooled in a 20 °C water bath for 3 min. Next, centrifugation was performed for 5 min to obtain the supernatant as above. Each test was also repeated in triplicate.

**Figure 6:**
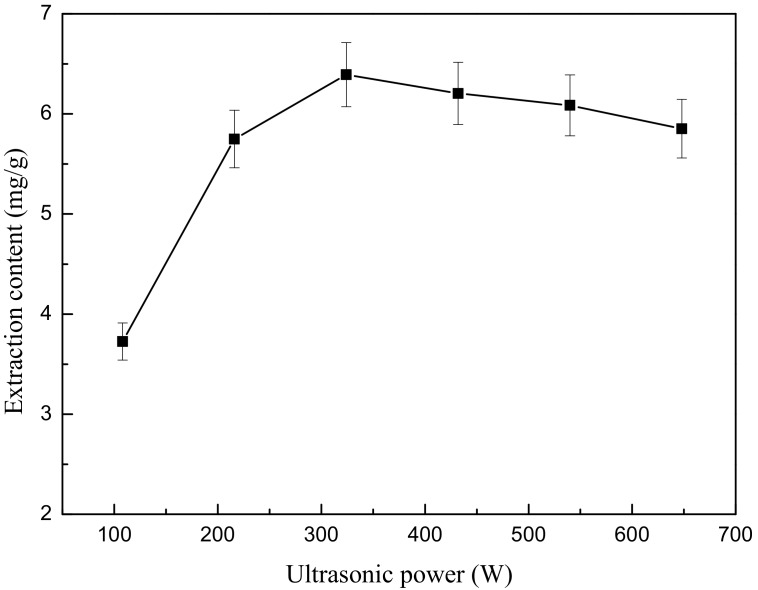
Effect of different power levels on ultrasonic extraction.

The effect of ultrasonic power is demonstrated in Figure 6. As seen in Figure 6, the increase in ultrasonic power ranging from 108 W to 324 W induces a significant increase in extraction content, with a maximum value of 6.392 mg/g. This is evidently due to the larger amplitude of the ultrasound waves travelling through the solvent. The higher the electrical power consumed, the more violently the bubbles collapse, and thus the more TF that can be extracted from the *O. fragrans* cells, while when the ultrasonic power increases from 324 W to 648 W, the yield of flavonoids will be decreased due to the degradation of flavonoids.

### 2.7. Effect of Ultrasonic Extraction Time on Extraction Yield of Total Flavonoids

First, 4.444 g weight of ground flower powder was added to 200 mL of 50% (*v*/*v*) ethanol. After the powder soaked in the liquid for 10 min, the mixture was treated by ultrasonic extractor at 378 W for various extraction times (5, 8, 11, 14, 17 and 20 min). After extraction, the mixture was cooled in a 20 °C water bath for 3 min. Next, centrifugation was performed for 5 min to obtain the supernatant as above. Each test was also repeated in triplicate.

**Figure 7:**
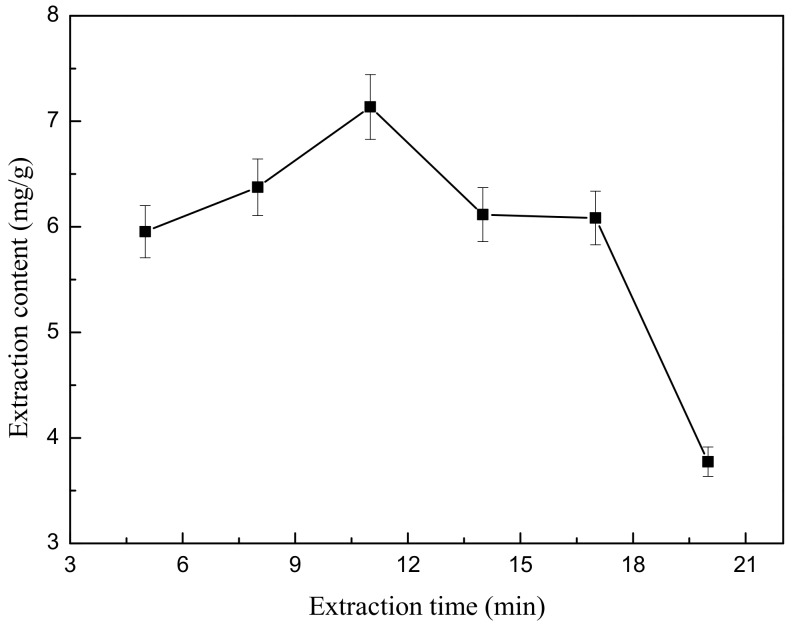
Effect of different treatment times on ultrasonic extraction.

The dependence between the value of extraction content and the ultrasonic extraction time is plotted in Figure 7. These data suggest that the ultrasonic extraction time has a significant effect on the extraction yield of total flavonoids. In ultra-sonication, microstreaming triggered by the collapse of the micro bubbles enhances the mass transfer.

### 2.8. Optimization of the Operation Parameters Using Response Surface Analysis

#### 2.8.1. Model Fitting and Statistical Analysis

In the current study, the Box-Behnken design with four factors and three levels, including five replicates at the centre point, was used. The variance and values of individual variables are given in Table 1. In Table 1, the experimental design and the results of 29 runs with BBD design are presented. The yields of total flavonoids affected by four factors were fitted with a second order polynomial equation and the values of the regression coefficients were calculated. The TFC value could be expressed by the following second order polynomial relationship in Equation (1):

(1) $$Y=7.69-0.32X_{1}+0.26X_{2}+0.39X_{3}+0.34X_{4}+0.30X_{1}X_{2}+0.24X_{1}X_{3}-0.37X_{1}X_{4}-0.35X_{2}X_{3}+0.013X_{2}X_{4}-0.23X_{3}X_{4}-0.96xX_{1}^{2}\;-0.23X_{2}^{2}-1.01X_{3}^{2}-0.44X_{4}^{2}$$

where Y represents the TFC and *X*_1_, *X*_2_, *X*_3_, *X*_4_ correspond to the ethanol concentration, microwave extraction time, ultrasonic extraction time and ultrasonic power, respectively. A summary of the analysis of variance (ANOVA) for the selected quadratic predictive model is shown in Table 2.

The correlation measure for testing the goodness-of-fit for the regression equation is the adjusted determination coefficient ($R_{Adj}^{2}$). The value of $R_{Adj}^{2}$ (0.9223) for Equation (2) is close to 1, which indicates a high degree of correlation between the actual and predicted values. The value of $R_{Adj}^{2}$ (0.9223) suggests that only approximately 7.77% of the total variation is not explained by the model:

(2) $$y\left(\mathrm{mg}/g\right)=\frac{m\times V}{M}$$

Here *m* (mg/mL) is the concentration of the extraction solution, *V* (mL) is the volume of the extracting solution and *M* (g) is the quality of the dried powder sample.

Table 2 shows that the model is highly significant with a low *p*-value (*p* < 0.0001). Furthermore, the *F*-value (24.73) is greater than the tabled critical *F*-value ($F_{0.05\left(14,4\right)}$ = 14.24) for fourteen and four degrees of freedom at the 95% confidence level, which indicates that the treatment differences are highly significant. The lack of fit of the model is insignificant relative to the pure error, which can be concluded from the lower calculated *F*-value (3.32) compared to the tabled critical *F*-value (4.65) for fourteen and ten degrees of freedom at the 95% confidence level. Thus, the model is effective to predict the experimental value within the range of variables employed.

The coefficient values of Equation (2) were calculated and tested to determine their significance using Design-Expert, and they are listed in Table 2. We found that (*X*_1_), (*X*_3_) and (*X*_4_) had the strongest effects on the response because the coefficients of *X*_1_, *X*_3_ and *X*_4_ were greater than that of *X*_2_. These factors exerted significant influences on the response. Table 2 shows that the linear coefficients (*X*_1_, *X*_2_, *X*_3_, *X*_4_), a quadratic term coefficient ($X_{1}^{2},X_{2}^{2},X_{3}^{2},X_{4}^{2}$), and a cross-product coefficient (*X*_1_*X*_2_, *X*_1_*X*_4_, *X*_2_*X*_3_) were significant with small *p* values at *p* < 0.05. The coefficient of the cross-product term (*X*_1_*X*_3_, *X*_3_*X*_4_) had a slight significance (*p* < 0.9).

#### 2.8.2. Response Surface Analysis and Optimization

The response data were analysed using the statistical software Design Design-Expert package. A TFC of 7.86 mg/g was obtained when the following optimum parameters were selected: ethanol concentration = 48.15%; microwave extraction time = 6.43 min; ultrasonic extraction time = 10.09 min; ultrasonic power = 370.9 W.

**Figure 8:**
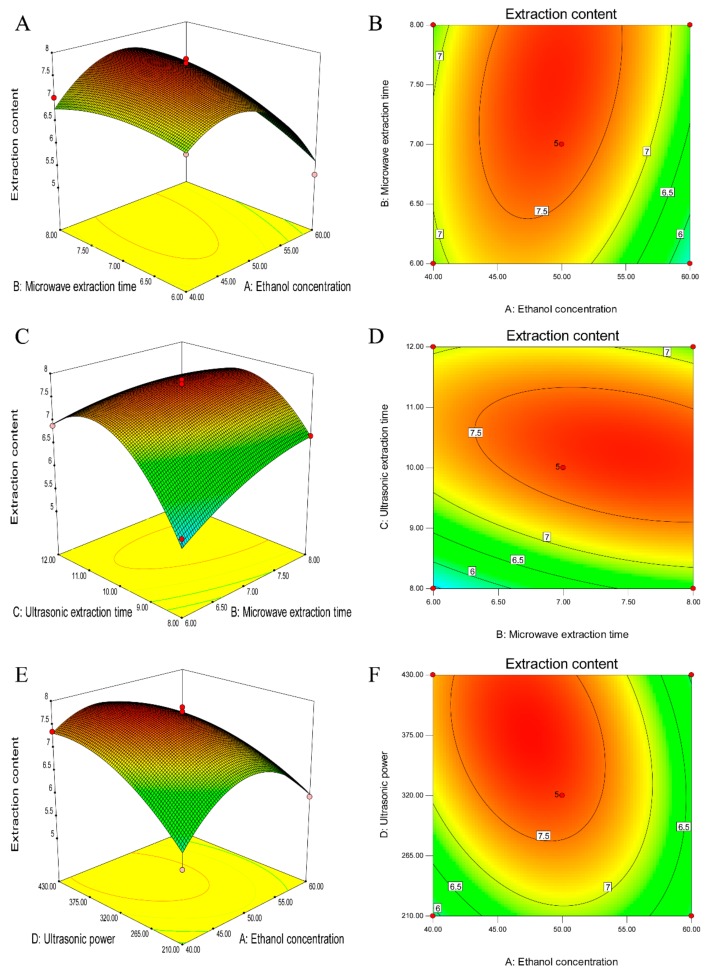
Effect of different microwave treatment times by different solvents on microwave extraction. (**A**) Response surface plot showing the extraction content as a function of microwave extraction time and ethanol concentration; (**B**) Contour plot showing the extraction content as a function of microwave extraction time and ethanol concentration; (**C**) Response surface plot showing the extraction content as a function of ultrasonic extraction time and microwave extraction time; (**D**) Contour plot showing the extraction content as a function of ultrasonic extraction time and microwave extraction time; (**E**) Response surface plot showing the extraction content as a function of ultrasonic power and ethanol concentration; (**F**) Contour plot showing the extraction content as a function of ultrasonic power and ethanol concentration. Figure 8E,F show how the TFC varied with different ethanol concentrations and ultrasonic power levels at a fixed microwave extraction time of 7 min and ultrasonic extraction time of 10 min. With a decrease in the ultrasonic power, the TFC decreased accordingly. An increase in the ethanol concentration from 40% (*v*/*v*) to 50% (*v*/*v*) resulted in an increase in the TFC.

The graphical representations were obtained by solving the RSM regression equation using Design Expert. In Figure 8, the response surfaces and contour plots show the effect of ethanol concentration, microwave extraction time, ultrasonic extraction time and ultrasonic power on the TF content. In each sub-figure, two other variables were maintained at the fixed points.

Figure 8A,B show the effect of the ethanol concentration and microwave extraction time on the TF content with an ultrasonic extraction time of 10 min and ultrasonic power level of 320 W. The TF content increased with an increasing ethanol concentration from 40% (*v*/*v*) to 47% (*v*/*v*) but decreased with an increasing microwave extraction time. For the variable microwave extraction time and ethanol concentration, they exerted significant effects on the TFC in both their individual and combined forms. These effects are clearly illustrated in the response surface in Figure 8A. 

The interaction of the microwave extraction time and ultrasonic time at a fixed ethanol concentration of 50% (*v*/*v*) and ultrasonic power of 320 W is shown in Figure 8C,D. The TFC increases with an increase in the ultrasonic time from 8 min to 10.7 min and slightly increases with an increase in the microwave time from 6 min to 8 min. Approximately 7.5 mg/g TFC was obtained with a microwave extraction time in the range of 5.3 min–7 min and a ultrasonic time in the range of 9.5 min–11.2 min. 

### 2.9. Model Validation

To confirm the accuracy of the model equation for the yield of total flavonoids, three verification experiments were performed under the optimal conditions. The average value of the experimental TFC yield was 7.85 mg/g, which was close to the predicated value. The result indicated no significant difference between the experimental and predicated values, and thus confirmed that the mathematical model developed by Box-Behnken design was accurate and adequate for predicating the extraction yield of total flavonoids. Furthermore, the optimized SC-MUAE presented a higher extraction content than those of MAE (4.789 mg/g), UAE (6.229 mg/g) and SC-UMAE (6.548 mg/g). 

## 3. Materials and Methods

### 3.1. Plant Material

Fresh flowers of *O. fragrans* were manually collected from Changguangxi National Wetland Park, Wuxi, China in October 2016. All samples were dried at room temperature for a month and stored in the laboratory (Room No. A206, the Jiangsu Key Laboratory of Advanced Food Manufacturing Equipment & Technology, Jiangnan University, Wuxi, China). First, the dried *O. fragrans* flowers were ground into powder by a high speed grinder (YF-111, Ruian Yongli Pharmaceutical Machinery, Ltd., Ruian, China). Then, the powder was passed through a 120 mesh sieve and the vacuum-packed samples were stored inside desiccators for further use in all experiments.

### 3.2. Reagents and Equipment

Ethanol was analytical reagent grade and purchased from Sino-pharm Chemical Reagent Co. Ltd. (Beijing, China). Deionized water was supplied by Logistic Division, Jiangnan University (Jiangsu, China). Rutin was purchased from the National Institute for Control of Pharmaceuticals and Biological Products (Beijing, China).

**Figure 9:**
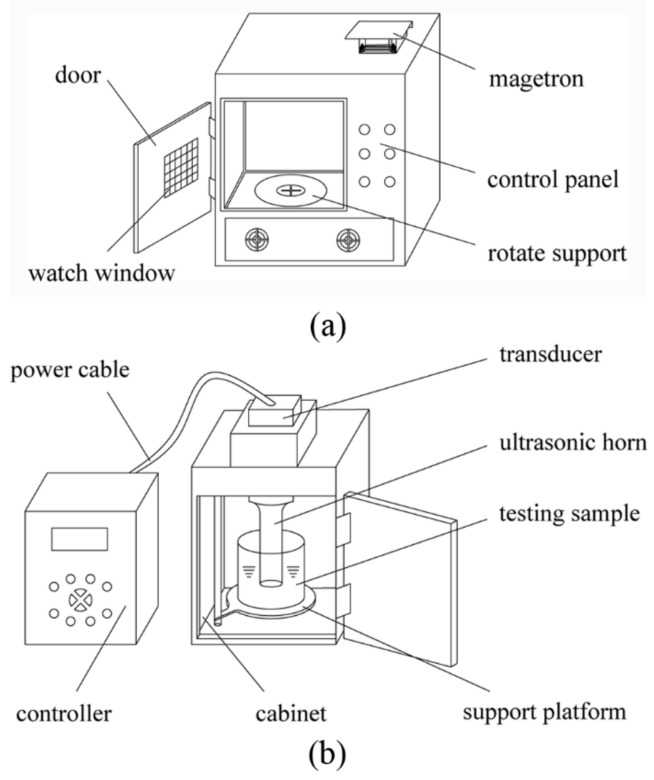
(**a**) Microwave reactor; (**b**) Ultrasonic extraction device.

A schematic diagram of the microwave extractor and ultrasonic extractor is depicted in Figure 9. The customized microwave extractor with inner capacity at 32 L was self-designed and placed in the Jiangsu Key Laboratory of Advanced Food Manufacturing Equipment and Technology (Wuxi, Jiangsu, China). The samples were irradiated on the rotated tray driven by a motor that held the samples to be treated in uniform microwave environment. A frequency of 2450 MHz was chosen for the microwave extractor, which was equipped with a digital timer to regulate the extraction time and thyristor power regulator to adjust the microwave output power (the latter was linearly adjusted from 0 W to 1000 W).

The ultrasound extractor (Model: JY99-IIDN) was provided by Ningbo Scientz Biotechnology Co. Ltd. (Ningbo, China), which had a maximum output power of 1800 W with a 20 kHz center frequency and a solid titanium probe of 25 mm diameter at 80% amplitude. Duty cycle of ultrasound pulse was set at 50% (2 s on, 2 s off).

The powder was weighed on an analytical balance (±0.1 mg) (AR 1140, Ohaus Corporation, Parsippany, NJ, USA). After the extraction, the solution of the resulting extract was transferred to a centrifuge tube for centrifugation at 12,000 rpm for 6 min by a high speed centrifuge (TGL-16C, Shanghai Anting Scientific Instrument Company, Shanghai, China). After centrifugation, the supernatants were collected and stored in ice water for further analysis.

### 3.3. Determination of Total Flavonoid Content

**Figure 10:**
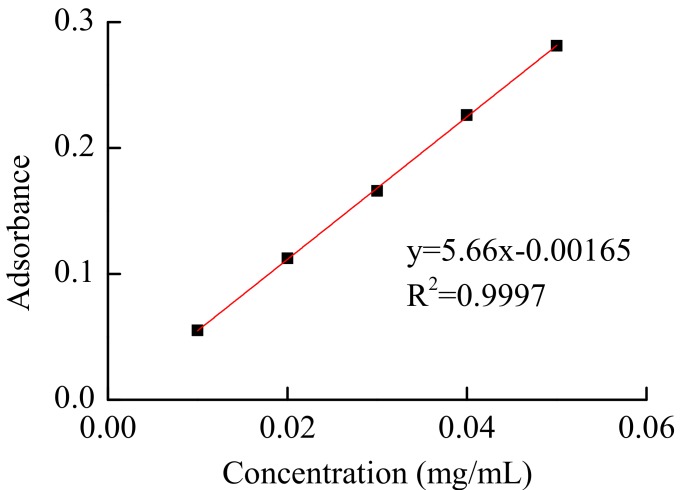
Standard curve of the concentration of total flavonoids.

The method to evaluate the total flavonoid content (TFC) was designed according to a modified chloride colorimetric assay [27]. Fifty mg of the rutin standard were dissolved into a 250 mL volumetric flask with ethanol as a stock solution. 0, 2, 4, 6, 8 and 10 mL of stock solution were transferred into separate 25 mL volumetric flasks. Subsequently, 1 mL NaNO_2_ with a concentration of 2% (*w*/*v*) and 1 mL Al(NO_3_)_3_ with a concentration of 10% (*w*/*v*) were added to each flask. The final volume of the mixed solution was adjusted to 25 mL with 50% (*v*/*v*) aqueous-ethanol. The absorbance was immediately measured against a blank at 510 nm, along with standards prepared similarly containing known rutin concentrations, with a UV spectrophotometer (UV-1800, Shimadzu, Kyoto, Japan). Triplicate analyses were conducted for each extract. A standard curve of TF was obtained as displayed in Figure 10. The amount of the total flavonoids is expressed as rutin equivalents through the standard calibration curve. The total flavonoid content (TFC) is defined on a weight basis as milligrams of rutin equivalents per gram of dry weight using Equation (2) [28]. Each assay was performed in triplicate.

### 3.4. Determination of Total Flavonoid Content

Four types of samples were prepared for a better visualization of the degree of internal cell disruption. One was initial powder that had not been treated by any extraction method, and the other three samples were treated by either microwave, ultrasound or the sequential combination of microwave and ultrasound.

Finally, a scanning electron microscope (Quanta-200, FEI Company, Hillsboro, OR, USA) with an accelerating voltage of 50 kV was applied for observing the microscopic changes of the cell surfaces.

### 3.5. Single-Factor Experimental Design

To study the influence of the critical variables on the yield of total flavonoids, single-factor experiments were performed and then the suitable ranges of variables were established. The solid-liquid ratios, ethanol concentration, microwave power, microwave extraction time, ultrasonic power and ultrasound extraction time were selected as the operating parameters in the single-factor experiments. To clearly determine the influence of each parameter, one operating parameter is varied while the other operating parameters are fixed during the single-factor experiments. Each experimental point was conducted in triplicate.

### 3.6. Response Surface Experimental Design

The single-factor experiments can help to determine, the preliminary range of the extraction variables for further processing optimization. Box-Behnken design-response surface methodology (BBD-RSM) was applied to optimize the SC-MUAE extraction conditions. Four independent variables were chosen for optimization: ethanol concentration (*X*_1_: 40–60%), microwave extraction time (*X*_2_: 6–8 min), ultrasonic extraction time (*X*_3_: 8–12 min) and ultrasonic power (*X*_4_: 210–430 W). The TFC (y, mg/g) was taken as the response of the design experiments. The coded levels (X*_i_*) of the independent variables were calculated according to the following equation:${\;X}_{i}=\left(x_{i}-x_{0}\right)/\Delta x$, where *x_i_* is the uncoded value of the independent variables, *x*_0_ is the uncoded value of the independent variable at the central point, and Δ*x* is the step change of variable. Four factors and three levels are shown in Table 3. Each experiment was repeated three times.

In response surface experiments, each testing sample was prepared by adding 4.444 g dried *O. fragrans* powder to 200 mL of 50% (*v*/*v*) aqueous-ethanol. The mixed solution was lightly agitated for 10 min before it was ready for further optimization experiments. In the response surface experiments, the output power of the microwave extractor was fixed at 465 W.

Twenty-nine combinations of the four variables were determined using Design-Expert software. A quadratic regression model was used to represent the response function as shown by the following Equation (3):

(3) $$Y=\beta_{o}+\overset{k}{\underset{i=1}{\sum }}\beta_{i}x_{i}^{2}+\overset{k}{\underset{i=1}{\sum }}\beta_{ii}x_{i}^{2}+\overset{k-1}{\underset{i=1}{\sum }}\overset{k}{\underset{j=2}{\sum }}\beta_{ij}x_{i}x_{j}$$

where Y, *β*_0_, *β_i_*, *β_ii_*, *β_ij_*, *k* are the response, regression coefficient for intercept, linear, quadratic, interaction terms and the number of studied factors, respectively. A statistical program package, Design Expert software (Version 8.0.6, Stat Ease Inc., Minneapolis, MN, USA) was applied for regression analysis of the experimental results obtained to determine the coefficient of regression equation.

### 3.7. Statistical Analysis

All experiments were performed in triplicate. Analysis of variance (ANOVA) of BBD test data was used to analyse the significance of factors and their interactions where *p* < 0.05 and *p* < 0.01 were regarded as significant and very significant, respectively. Single-factor experimental results were statistically evaluated by SPSS (Version 19.0, IBM Corporation, Armonk, NY, USA) in order to detect significant differences among values. Significant differences at the level of 5% (*p* < 0.05) were analysed by statistical *F*-test.

## 4. Conclusions

Extraction is the key step for the comprehensive utilization of total flavonoids from the *O. fragrans* flower. Compared with traditional extraction methods, SC-MUAE is a more effective process to extract total flavonoids from the *O. fragrans* flower. The results here can be exploited to develop TF extraction systems for future industrial purposes. From the results of this study, we can state the following conclusions:

(1) The single-factor experiments revealed that the solid-liquid ratio, ethanol concentration, microwave extraction time, ultrasonic power and ultrasonic extraction time have significant effects on the yield of total flavonoids.
(2) The yield of total flavonoids was modelled using a response surface methodology. The optimum extraction conditions were as follows: ethanol concentration (48.15%), microwave extraction time (6.43 min), ultrasonic extraction time (10.09 min) and ultrasonic power (370.9 W). Under these conditions, the extraction yield of TF was 7.86 mg/g.
(3) SC-MUAE has a higher extraction yield of TF from *O. fragrans* flowers than MAE, UAE and SC-UMAE. The experimental results demonstrated that SC-MUAE has potential to be applied in large-scale extractions of TF from *O. fragrans* flowers.

## Figures and Tables

**Table 1:** Box-Behnken experimental design with the independent variables.

Run	Coded Variable Levels				Concentration
	$X_{1}$	$X_{2}$	$X_{3}$	$X_{4}$	
1	1	−1	0	0	5.301
2	0	0	0	0	7.777
3	0	0	−1	−1	5.371
4	0	−1	−1	0	5.622
5	−1	0	0	−1	5.543
6	0	1	0	1	7.614
7	0	0	1	1	6.572
8	−1	1	0	0	7.031
9	1	0	0	−1	5.941
10	0	0	0	0	7.841
11	−1	0	0	1	7.355
12	0	0	0	0	7.653
13	−1	0	−1	0	5.855
14	0	0	−1	1	6.181
15	0	−1	0	1	7.161
16	0	0	0	0	7.607
17	1	0	−1	0	4.727
18	−1	0	1	0	6.321
19	−1	−1	0	0	6.829
20	0	0	0	0	7.518
21	1	0	1	0	6.167
22	0	1	1	0	6.537
23	1	0	0	1	6.257
24	0	0	1	−1	6.665
25	0	1	0	−1	6.944
26	0	−1	0	−1	6.543
27	1	1	0	0	6.698
28	0	1	−1	0	6.677
29	0	−1	1	0	6.896

**Table 2:** Test of significance for regression coefficient.

Source	Sum of Squares	Df	Mean Square	*F*	*p*
Model	17.96	14	1.28	24.73	<0.0001
*X*_1_—ethanol concentration	1.23	1	1.23	23.74	0.0002
*X*_2_—microwave extraction time	0.83	1	0.83	15.94	0.0013
*X*_3_—ultrasonic extraction time	1.86	1	1.86	35.87	<0.0001
*X*_4_—ultrasonic power	1.42	1	1.42	27.42	0.0001
*X*_1_*X*_2_	0.36	1	0.36	6.88	0.0200
*X*_1_*X*_3_	0.24	1	0.24	4.57	0.0506
*X*_1_*X*_4_	0.56	1	0.56	10.77	0.0055
*X*_2_*X*_3_	0.50	1	0.50	9.64	0.0078
*X*_2_*X*_4_	7.022 × 10^−4^	1	7.022 × 10^−4^	0.014	0.9090
*X*_3_*X*_4_	0.20	1	0.20	3.95	0.0669
*X*_1_^2^	5.93	1	5.93	114.39	<0.0001
*X*_2_^2^	0.34	1	0.34	6.49	0.0232
*X*_3_^2^	6.63	1	6.63	127.83	<0.0001
*X*_4_^2^	1.27	1	1.27	24.40	0.0002
Residual	0.73	14	0.052		
Lack of fit	0.65	10	0.065	3.32	0.1293
Pure error	0.078	4	0.020		
Corrected total	18.68	28			
		*R*^2^ = 0.9611; $R_{adj}^{2}=0.9223$; C.V.% = 3.45			

**Table 3:** Independent variables and their levels in the response surface design.

Independent Variables	Symbol	Levels		
		−1	0	1
Ethanol concentration (%)	$x_{1}$	40	50	60
Microwave extraction time (min)	$x_{2}$	6	7	8
Ultrasonic extraction time (min)	$x_{3}$	8	10	12
Ultrasonic power (W)	$x_{4}$	210	320	430
